# Acetic Acid-Induced Ulcerative Colitis in Sprague Dawley Rats Is Suppressed by Hydroethanolic Extract of *Cordia vignei* Leaves through Reduced Serum Levels of TNF-*α* and IL-6

**DOI:** 10.1155/2020/8785497

**Published:** 2020-02-06

**Authors:** George Owusu, David D. Obiri, George K. Ainooson, Newman Osafo, Aaron O. Antwi, Babatunde M. Duduyemi, Charles Ansah

**Affiliations:** ^1^Department of Pharmacology, Faculty of Pharmacy and Pharmaceutical Sciences, College of Health Sciences, Kwame Nkrumah University of Science and Technology (KNUST), Kumasi, Ghana; ^2^Department of Pharmacology, School of Medicine and Health Sciences, University for Development Studies, Tamale, Ghana; ^3^Department of Pathology, School of Medical Sciences, Kwame Nkrumah University of Science and Technology (KNUST), Kumasi, Ghana

## Abstract

**Background:**

Ulcerative colitis (UC) is a recurrent inflammatory bowel disease (IBD) that causes long-lasting inflammation on the innermost lining of the colon and rectum. Leaf decoctions of *Cordia vignei* have been used in traditional medicine either alone or in combination with other plant preparations to treat the disease.

**Aim:**

In this study, we investigated the effect of hydroethanolic extract of *Cordia vignei* have been used in traditional medicine either alone or in combination with other plant preparations to treat the disease.

**Method:**

Male Sprague Dawley rats received oral treatment of either saline (10 ml/kg), sulfasalazine (500 mg/kg), or CVE (30-300 mg/kg) daily for 7 days. On day 4, colitis was induced by a single intrarectal administration of 500 *μ*l of acetic acid (4% *v*/*v*/

**Results:**

CVE significantly (*P* < 0.05) prevented colonic ulceration and reduced the inflammatory score. Serum levels of TNF-*α* and IL-6 were significantly reduced. Depletion of superoxide dismutase (SOD) and catalase (CAT) activities by acetic acid was significantly inhibited while lipid peroxidation indexed as malondialdehyde (MDA) level in the colon was reduced. However, loss of body weight was not significantly affected by treatment with CVE.

**Conclusion:**

This data suggest that CVE has a potential antiulcerative effect.

## 1. Introduction

Ulcerative colitis is a recurrent inflammation of the colon. The clinical symptoms often presented by this disease are diarrhoea, loss of weight, nausea, and abdominal pain which affect quality of life [1]. The pathological features include inflammatory cell infiltration and activation, downstream expression of nuclear factor kappa B- (NF-*κ*B-) dependent proinflammatory mediators such as tumor necrosis factor alpha (TNF-*α*) and interleukin 6 (IL-6), excessive generation of free radicals such as reactive oxygen species (ROS) and reactive nitrogen species (RNS), depletion of antioxidant capacity of the colon, and loss of mucosal integrity [2]. Although genetic, immunological, and environmental factors are implicated in the pathogenesis of ulcerative colitis, the exact aetiology of the disease still remains elusive [3]. Studies in this area contribute to the attempt to elucidate the causes and development of the disease. Also, the increasing number of cases of ulcerative colitis shows the importance of searching for new compounds that can control or eliminate the disease that severely affects the quality of life of these patients.

Due to resemblance of experimentally induced colitis in animals and ulcerative colitis in humans, the former has become an indispensable tool to study the pathomechanism of the disease and also for screening of novel compounds for their antiulcerative activity [4]. An acetic acid-induced ulcerative colitis model involving intrarectal administration of acetic acid in rats was employed in this study. Currently, conventional drugs that are commonly used in the treatment of colitis include 5-aminosalicylic acid, systemic corticosteroids, immunomodulators, and vitamin E [5]. Since excessive production of reactive oxygen species seems to play an important role in tissue damage and inflammatory process of the disease, administration of natural substances as treatment may be a valuable option for new therapies [6–8]. One plant of interest is *Cordia vignei*. It is a woody plant belonging to the family Boraginacaea. It grows to about 30 cm in diameter and 10 m tall. In traditional medicine, decoctions of the leaves of the plant are used either alone or in combination with other plants to treat inflammatory disorders [9]. An ethnopharmacological survey conducted by Agyare et al. (2017) revealed that the leaves of the plant are traditionally used in Ghana for treatment of prostate cancer [10]. Despite the usefulness of the plant in traditional medicine, scientific investigation of its effects has not been conducted.

This study evaluates the antiulcerative effect of hydroethanolic leaf extract of *Cordia vignei* in rats to provide a scientific data and validate the traditional use. We also determined whether the underlying mechanisms involve protection of antioxidant potential of the colon and/or reduction of the serum levels of principal proinflammatory mediators such as TNF-*α* and IL-6.

## 2. Materials

### 2.1. Plant Collection and Extraction

Fresh leaves of *Cordia vignei* were collected from the Diabaa Forest Reserve (located at longitudes 3° W and 3° 30′ W and latitudes 7° N and 7° 30′ N) in the Dormaa West District of Brong Ahafo region of Ghana. The leaves were authenticated at the herbarium of the Department of Herbal Medicine, Faculty of Pharmacy and Pharmaceutical Sciences, Kwame Nkrumah University of Science and Technology (KNUST), Kumasi, Ghana. A voucher specimen (KNUST/HM1/2017/L003) was kept at the herbarium. The plant material was air dried for seven days. Three and half kilograms (3.5 kg) of the dried material was pulverized into fine powder using a heavy-duty blender (37BL85 (240CB6) Waring Commercial, USA). The fine powder was macerated with 5 l of 70% (*v*/*v*) ethanol for 72 h, filtered and concentrated in a rotary evaporator (Rotavapor R-210, BUCHI, Switzerland) at 50°C, and further solidified in an oven (Gallenkamp OMT Oven, Sanyo, Japan). The moist gummy solid extract with a total yield of 11.62% (*w*/*w*) was kept in a desiccator for 72 h. For oral administration, the extract was reconstituted as an emulsion in 2% tragacanth and referred to as *Cordia vignei* extract (CVE) in this study.

### 2.2. Animals

Male Sprague Dawley rats (9-11 weeks old, weighing 200-220 g) were obtained from the Animal House of the Department of Pharmacology, KNUST, and maintained under standard laboratory conditions. All animals were carefully handled in accordance with National Institute of Health Guidelines for the Care and Use of Laboratory Animals (NIH, Department of Health and Human Services; Publication No. 85-23, revised 2011) [11]. All protocols involving the use of animals were approved by the Animal Ethics Committee of Kwame Nkrumah University of Science and Technology.

### 2.3. Drugs and Chemicals

Sulfasalazine (KAR LABS LTD, New Delhi, India) and ELISA Kit for rat TNF-*α* and IL-6 (Boster Biological Technology Ltd., CA, USA), acetic acid, and ethyl alcohol (British Drug House, Poole, UK) were purchased.

## 3. Method

### 3.1. Induction of Colitis

Rats were randomly selected into 6 groups (*n* = 5) and received daily oral administration of either normal saline (10 ml/kg), sulfasalazine (500 mg/kg), or CVE (30, 100, and 300 mg/kg) from day 0 to day 7. On day 4, colitis was induced by intrarectal administration of 500 *μ*l of acetic acid (4% *v*/*v*). Body weights of rats were taken daily. Rats were euthanized on day 8 by cervical dislocation under anaesthesia with 50 mg/kg pentobarbitone (i.p.).

#### 3.1.1. Assessment of Colon Weight-to-Length Ratio

Rats were dissected and their colons were extirpated, opened longitudinally, and rinsed gently under running water to remove the faeces. Colons were placed on nonabsorbent surfaces and weight-to-length ratios were blindly assessed. Extirpated colons were stored at –80°C.

#### 3.1.2. Macroscopic Assessment of Colonic Damage

Macroscopically visible injuries such as thickening, shortening, hyperemia, and necrosis were blindly scored from 0-100% based on increasing order of severity as described by Ballester et al. [12] and Motavallian-Naeini et al. [13]. The mean scores were presented as the Disease Activity Index (DAI) of the colon.

#### 3.1.3. Microscopic Assessment of Colonic Damage

Samples of the distal colons were fixed immediately in 10% formaldehyde, embedded in liquid paraffin, cut into transverse sections of 5 *μ*m thick using a Leica RM 2125 Microtome (Leica Biosystems, Wetzlar, Germany), and then mounted on glass slides and stained with haematoxylin and eosin (H&E). Microscopic changes such as necrosis, fibrosis, hyperemia, epithelial damage, ulceration, infiltration, and submucosal abscesses were scored on a 0-4 scale where 0 denotes no detectable damage and 4 denotes most severe damage [1].

#### 3.1.4. Assessment of Antioxidant Enzymes in the Colon

Colons were homogenized using a Potter-Elvehjem homogenizer (Ultra-Turrax T25, Janke and Kunkel IKA-Labortechnik, Staufen, Germany) on ice-cold Tris-HCl buffer (0.01 M, pH 7.4) to give a 10% homogenate which was used for assays of superoxide dismutase and catalase activity in the colons as described below.

*(1) Superoxide Dismutase (SOD)*. Activity of SOD in the colon was determined as described by Misra and Fridovich (1972) [14] with a slight modification. Briefly, 750 *μ*l ethanol (96% *v*/*v*) and 150 *μ*l of ice-cold chloroform were added to 500 *μ*l of the tissue homogenate and centrifuged at 2000 rpm for 20 min at 25°C. To 500 *μ*l of the supernatant, 500 *μ*l EDTA (0.6 mM) and 1 ml carbonate bicarbonate buffer (0.1 M, pH 10.2) were added. The reaction was initiated by the addition of 50 *μ*l of adrenaline (1.3 mM). Absorbance was measured against a blank at 480 nm for 4 min using the Cecil ultraviolet-visible spectrophotometer (CE 2041, Milton, England).

Percentage inhibition of autoxidation of adrenaline was calculated using the formula:

(1) $$\begin{array}{c} \text{Percentage inhibition}=\left[\frac{{\text{Absorbance}}_{\text{test}}-{\text{Absorbance}}_{\text{blank}}}{{\text{Absorbance}}_{\text{test}}}\right]\times 100. \end{array}$$

The enzyme activity was expressed as unit per mg protein. One unit of the enzyme activity was defined as the amount of enzyme that inhibits the autooxidation of adrenaline by 50% at 25°C.

(2) $$\begin{array}{c} \text{Unit of SOD activity}/\text{mg protein}=\left[\frac{\%\text{ inhibition}}{50\times \text{weight of protein}}\right]\times 100. \end{array}$$

*(2) Catalase*. Activity of catalase in the colon was determined by using the procedure described by Sinha [15] with a slight modification. The assay mixture containing 1 ml (0.01 M) phosphate buffer (pH 7.0), 500 *μ*l H_2_O_2_ (1.18 M), and 400 *μ*l of water was added to 100 *μ*l of the aliquot of the tissue supernatant and incubated at 28°C for 5 min to initiate the reaction. The reaction was terminated by adding 2 ml acetic acid-dichromate mixture comprising a 3 : 1 ratio of glacial acetic acid and 5% potassium dichromate. Absorbance of the chromic acetate formed was measured at 620 nm with Cecil ultraviolet-visible spectrophotometer (CE 2041, Milton, England). One unit of catalase activity was defined as the amount of enzyme needed to cause decomposition of 1 *μ*mol H_2_O_2_ per min per mg protein at 25°C and pH 7.0. The enzyme activity was expressed in terms of its molar extinction coefficient of 39.4 M^−1^ cm^−1^:

(3) $$\begin{array}{c} \text{mUnitCAT mg}/\text{protein}=\left[\frac{{\text{Absorbance}}_{620\text{ nm}}}{3.94\times \text{weight of protein}}\right]\times 1000. \end{array}$$

*(3) Malondialdehyde (MDA) Level*. Levels of malondialdehyde a byproduct of lipid peroxidation in the colon was estimated as described by Heath and Parker [16]. Briefly, 1 ml of the tissue extract was added to a 3 ml mixture of 20% trichloroacetic acid (TCA) and 0.5% thiobarbituric acid (TBA). The mixture was heated at 95°C for 30 min, cooled in an ice bath, and centrifuged at 2,000 rpm for 10 min. Absorbance of the MDA-TBA complex formed was read at 532 nm against the blank using a UV mini-1240 single beam spectrophotometer (Shimadzu Scientific Instrument, SSI, Kyoto, Japan). The concentration (nmol/mg protein) of MDA was calculated using the MDA extinction coefficient of 1.56 × 10^−5^ M^−1^ cm^−1^.

### 3.2. Assay of Serum TNF-*α* and IL-6

Colitis was induced in rats as described earlier and the rats were euthanized on day 8. Blood samples were collected in clot activator tubes (Add Surgifield Medicals, Middlessex, England) and centrifuged (Heraeus Megafuge 16R, Thermo Scientific) at 3000 rpm for 30 min at 4°C to obtain the serum. The serum was assayed quantitatively for TNF-*α* and IL-6 by using their respective Picokine ELISA kits as instructed by the manufacturer.

### 3.3. Haematology

Blood samples were also collected into vacutainer sterile tubes coated with EDTA as an anticoagulant. Full blood count was conducted using the Automated Cell Analyzer (YSTE880-Guangzhou Yueshen Medical Equipment Co. Ltd., China).

### 3.4. Data Analyses

Body weight change values were normalized as percentage of the body weight taken at the start of the experiment. Time-course curves for body weight were subjected to two-way (treatment × time) ANOVA followed by Bonferroni's post hoc test. GraphPad Prism for Windows version 6.01 was used for all statistical analyses and plotting of graphs. Results were presented as mean ± SEM. *P* < 0.05 was considered statistically significant.

## 4. Results

### 4.1. Effect of Hydroethanolic Leaf Extract of *Cordia vignei* on Body Weight of Acetic Acid-Induced Colitic Rats

**Figure 1:**
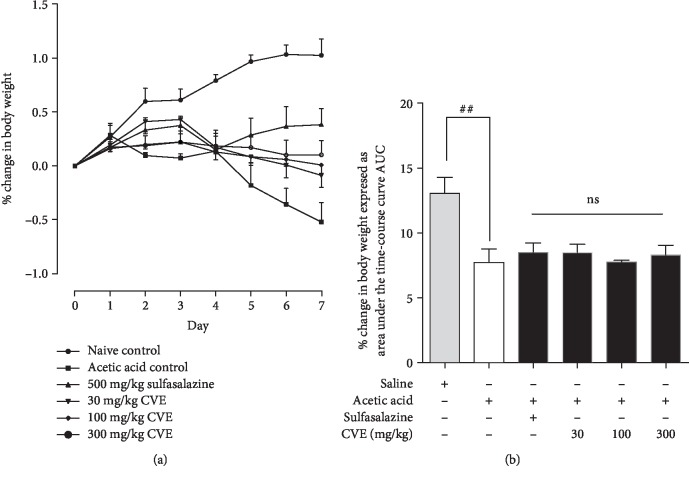
Effect of hydroethanolic leaf *Cordia vignei* extract on the body weights of acetic acid-induced colitic rats. Rats received daily oral administration of normal saline (10 ml kg^−1^), sulfasalazine (500 mg kg^−1^), or CVE (30-300 mg kg^−1^) for 7 days. On day 4, rats were challenged with 500 *μ*l acetic acid. Changes in weight were calculated and presented as *mean* ± *SEM*.^##^*P* < 0.005 (acetic acid control *vs*. naïve control), ns: not significant (acetic acid control vs. treatment group) using *t* test.

Intrarectal injection of acetic acid in rats evoked colonic inflammatory response which became evident as passing of loose stool (with or without occult blood) and loss of body weight. The naïve control rats showed a gradual increase in weight (0-1.07%) throughout the 7 days (Figure 1(a)). In contrast to the naïve control, acetic acid control rats exhibited decrease in body weight from day 2 to day 7 (Figure 1(a)). Body weights of sulfasalazine-treated rats decreased from day 3 to day 4 and then slightly increased from day 5 to day 7 (Figure 1(a)). On the other hand, body weights of CVE-treated rats slightly declined from day 3 to day 7 (Figure 1(a)).

The total change in body weight of rats over the 7 days was calculated as area under the curve (AUC) as shown in Figure 1(b). The AUC for the naïve control over the 7 days was 12.57 ± 1.079. Acetic acid significantly reduced the AUC to 7.495 ± 0.8371 (*P* = 0.0059) compared to naïve control (Figure 1(b)). The AUC of sulfasalazine-treated rats was 8.956 ± 0.7331 (*P* = 0.2258) compared to acetic acid control while the AUC of CVE-treated rats were 8.257 ± 0.56 (*P* = 0.4700), 7.858 ± 0.14 (*P* = 0.6800), and 8.086 ± 0.63 (*P* = 0.5885), respectively, at 30, 100, and 300 mg kg^−1^ compared to acetic acid control (Figure 1(b)).

### 4.2. Effect of Hydroethanolic Leaf Extract of *Cordia vignei* on Colon Weight-to-Length Ratio of Acetic Acid-Induced Colitic Rats

**Figure 2:**
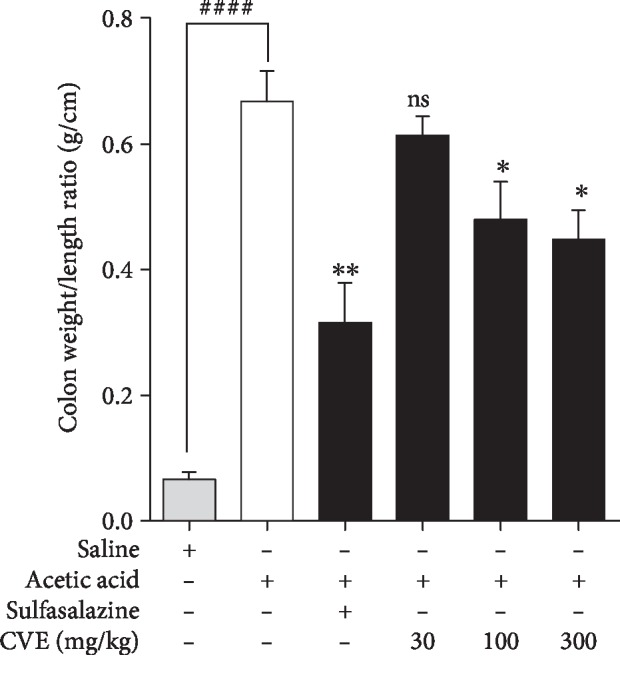
Effect of *Cordia vignei* leaf extract on colon weight/length ratio of acetic acid-induced colitic rats. Colitis was induced in rats as described in Method. Rats were sacrificed on day 8 and colon weight-to-length ratios were assessed. Results were presented as mean ± SEM. ^####^*P* < 0.0001 (acetic acid control *vs.* naïve control); ^∗^*P* < 0.05, ^∗∗^*P* < 0.01, and ns not significant (acetic acid control *vs.* treatment group) using *t* test.

Increase in inflammatory cell infiltration, vascular permeability, and oedema of the colon contributes to the increase in weight/length ratio of the colon [17]. The naïve control rats had mean colon weight/length ratio of 0.07 ± 0.01 g/cm which was significantly increased to 0.67 ± 0.05 (*P* = 0.0001) in the acetic acid control rats (Figure 2). Sulfasalazine reduced this mean colon weight/length ratio to 0.32 ± 0.06 g/cm (*P* = 0.0022) compared to acetic acid control rats. Similarly, CVE significantly reduced the mean colon weight/length ratio to 0.48 ± 0.06 (*P* = 0.0411) and 0.45 ± 0.05 g/cm (*P* = 0.0116), respectively, at doses 100 and 300 mg kg^−1^ albeit insignificant (0.6 ± 0.03; *P* = 0.3740) at 30 mg/kg (Figure 2).

### 4.3. Effect of Hydroethanolic Leaf Extract of *Cordia vignei* on Macroscopic Score of Colons of Acetic Acid-Induced Colitic Rats

**Figure 3:**
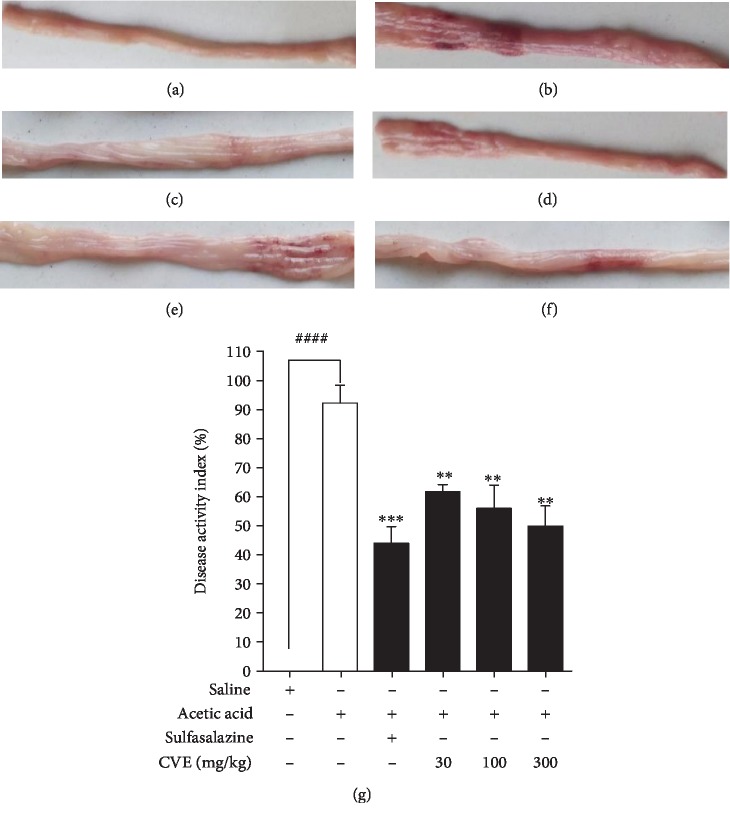
Effect of hydroethanolic leaf extract of *Cordia vignei* on macroscopic score of colons of acetic acid-induced colitic rats. Colitis was induced as described in Method. Rats were euthanized on day 8 and the colons were excised, longitudinally opened, washed, and examined (a–f). Colons were scored from 0-100% in ascending order of severity of inflammation (g). Results were presented as mean ± SEM. ^####^*P* < 0.0001 (acetic acid control *vs.* naïve control); ^∗∗^*P* < 0.01; ^∗∗∗^*P* < 0.001 (acetic acid control *vs.* treatment group) using *t* test.

The naïve control rats did not show any sign of colitis (Figure 3(a)). Intrarectal administration of acetic acid evoked a colonic inflammation characterized by increased neutrophil infiltration, massive necrosis of mucosal and submucosal layers, submucosal ulceration, increase in vascular dilation, and oedema (Figure 3(b)). In contrast to the disease control rats, sulfasalazine-treated rats showed mild colonic inflammation with less infiltration and ulceration (Figure 3(c)). Similarly, rats treated with CVE exhibited moderate colonic inflammation with less infiltration and ulceration as shown in Figures 3(d)–3(f), respectively, for 30–300 mg kg^−1^.

Visible signs of colonic inflammation such as thickening, shortening, hyperemia, and necrosis were quantified as a Disease Activity Index (DAI) ranging between 0 and 100% in ascending order of severity (Figure 3(g)). Naïve control rats had DAI of 0%. In contrast to the naïve control rats, acetic acid control rats showed very severe colonic inflammation characterized by hyperemia, thickening, shortening, and oedema with DAI of 92.26 ± 6.09%. Sulfasalazine significantly reduced the DAI to 44.00 ± 5.81% while the extract significantly and dose dependently protected the rats from severe colonic ulceration. DAI of the CVE-treated groups were 61.78 ± 2.46%, 55.98 ± 8.08%, and 49.288 ± 6.95%, respectively, administered at 30, 100, and 300 mg/kg^−1^ CVE (Figure 3(g)).

### 4.4. Effect of *Cordia vignei* Leaf Extract on Colon Histopathology of Acetic Acid-Induced Colitic Rats

**Figure 4:**
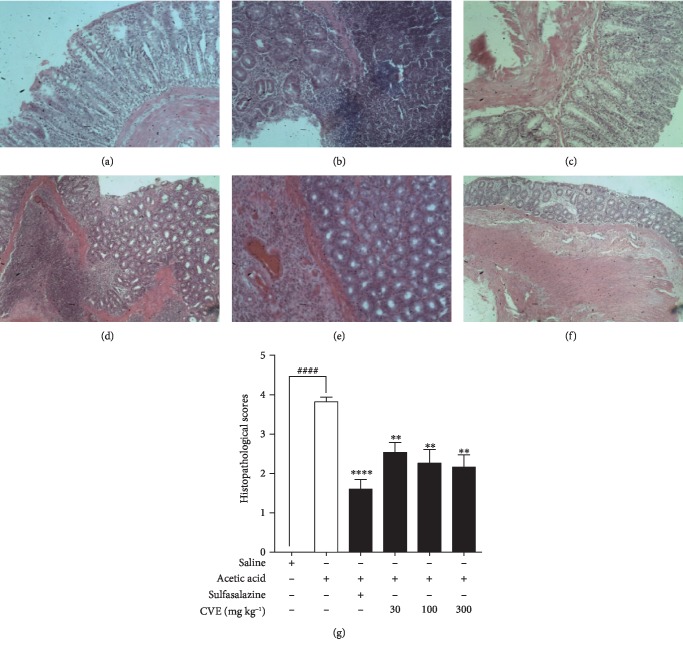
Effect of hydroethanolic extract of *Cordia vignei* leaves on microscopic changes in the colons of acetic acid-induced colitic rats. Colitis was induced as described in Method. Colons were fixed in 10% formaldehyde, sectioned (5 *μ*m thick), and stained with H&E. Representative photomicrographs shown are colons of naïve control rats (a), acetic acid control rats (b), sulfasalazine-treated rats(c), and 30-300 mg kg^−1^ CVE-treated rats (d–f, respectively). Microscopic changes were quantified based on severity of damage (*g*). Results were presented as mean ± SEM. ^####^*P* < 0.0001 (acetic acid control vs. naïve control); ^∗∗^*P* < 0.01; ^∗∗∗∗^*P* < 0.0001 (acetic acid control vs. treated group).

Colons of naïve control rats exhibited no observable histopathological changes (Figure 4(a)). Conversely, colons of acetic acid control rats showed necrosis and abscesses in the colonic mucosa with infiltration of inflammatory cells into the submucosa (Figure 4(b)). Treatment of rats with sulfasalazine significantly prevented the colonic injury. There was mild infiltration and abscesses (Figure 4(c)). CVE-treated rats' colons exhibited reduced colonic inflammation. There was mild infiltration and abscesses in the mucosa. Epithelial cell loss and gross colonic injury were significantly reduced at doses 30-300 mg kg^−1^ (Figure 4(d)–4(f)).

On quantification, the mean histopathological score of the colons of naïve control rats was 0.0 ± 0.0 (Figure 4(g)). In contrast to the naïve control, colons of acetic acid control rats exhibited severe inflammation with a significant increase in the histopathological score of 3.820 ± 0.12 (*P* = 0.0001). Sulfasalazine significantly reduced this mean score to 1.604 ± 0.24 (*P* = 0.0001) while CVE treatment ameliorated acetic acid-induced colon damage and significantly reduced inflammatory score to 2.54 ± 0.26 (*P* = 0.0019), 2.26 ± 0.35 (*P* = 0.0030), and 2.162 ± 0.32 (*P* = 0.0030) at doses 30, 100, and 300 mg kg^−1^, respectively, when compared to acetic acid control (Figure 4(g)).

### 4.5. Effect of Hydroethanolic Extract of *Cordia vignei* Leaves on Activity of Superoxide Dismutase and Catalase on Colons of Acetic Acid-Induced Colitic Rats

#### 4.5.1. Superoxide Dismutase

**Figure 5:**
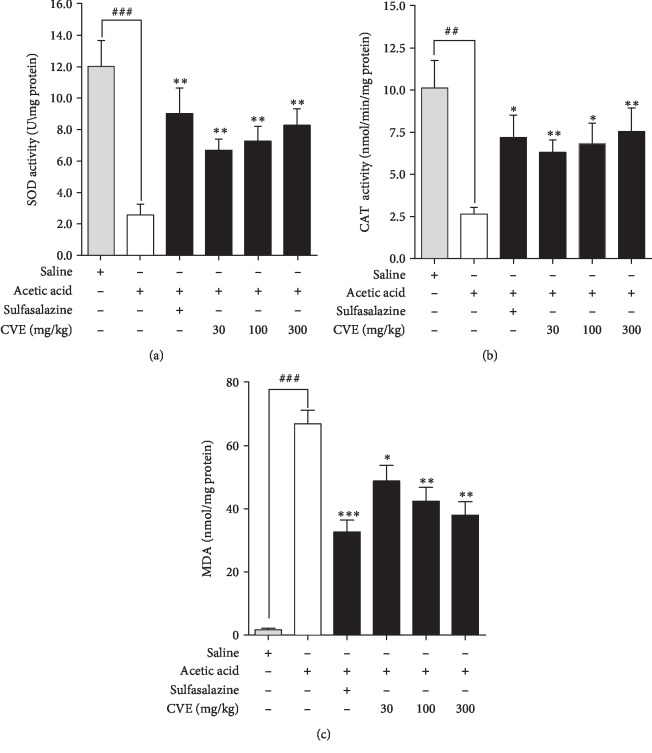
Effect of *Cordia vignei* leaf extract on SOD, CAT, and MDA on the colons of acetic acid-challenged rats. Rats (*n* = 5) orally received normal saline (10 ml kg^−1^), sulfasalazine (500 mg kg^−1^), or CVE (30-300 mg kg^−1^) daily for 7 days. On day 4, rats were challenged with 500 *μ*l acetic acid (4% *v*/*v*). Rats were sacrificed on day 8 and the colons were homogenized and centrifuged. The supernatant was used for the assay of activity of SOD (a) and CAT (b) and levels of MDA (c). Results were presented as mean ± SEM and statistically compared using student's *t* test. ^####^*P* < 0.0001;^###^*P* < 0.001;^##^*P* < 0.01 (acetic acid control *vs*. naïve control); ^∗^*P* < 0.05; ^∗∗^*P*, <0.01, ^∗∗∗^*P* < 0.001 (acetic acid control *vs.* treatment group).

The mean activity of superoxide dismutase (SOD) in the tissue of naïve animals was 12.02 ± 1.65 U/mg protein (Figure 5(a)). Intrarectal administration of acetic acid significantly reduced the mean SOD activity (U/mg protein) in the tissue to 2.55 ± 0.07 U/mg (*P* = 0.0007). Sulfasalazine significantly increased SOD activity (U/mg protein) to 9.02 ± 1.62 (*P* = 0.0063). Similarly, treatment of rats with CVE significantly increased the mean SOD activity (U/mg protein) to 6.68 ± 0.72 (*P* = 0.0035), 7.26 ± 0.94 (*P* = 0.0038), and 8.28 ± 1.05 (*P* = 0.00190, respectively, at doses 30, 100, and 300 mg kg^−1^ (Figure 5(a)).

#### 4.5.2. Catalase

The mean CAT activity (nmol/min/mg protein) of naive control rats was 10.12 ± 1.64 (Figure 5(b)). Acetic acid significantly reduced CAT activity to 2.64 ± 0.39 nmol/min/mg protein (*P* = 0.0022). Sulfasalazine significantly increased the mean CAT activity (nmol/min/mg protein) to 7.20 ± 1.33 (*P* = 0.0110) compared to the acetic acid control group. Treatment of rats with 30, 100, and 300 mg kg^−1^ CVE significantly increased CAT activity (nmol/min/mg protein) to 6.30 ± 0.74 (*P* = 0.0023), 6.82 ± 1.23 (*P* = 0.0119), and 7.54 ± 1.41 (*P* = 0.0099), respectively, (Figure 5(b)).

#### 4.5.3. Malondialdehyde (MDA) Levels

There was an insignificant mean MDA level of 1.68 ± 0.58 nmol/mg protein in naïve control rats (Figure 5(c)). Acetic acid significantly increased the mean MDA level to 66.86 ± 4.32 nmol/mg protein (*P* = 0.0001) compared to the naïve control. Sulfasalazine decreased the mean MDA level to 32.64 ± 3.78 nmol/mg protein (*P* = 0.0003) compared to acetic acid control. CVE also reduced the mean MDA level to 48.79 ± 4.93 (*P* = 0.0247), 42.41 ± 4.37 (*P* = 0.0041), and 38.03 ± 4.26 (*P* = 0.0014) nmol/mg protein at 30, 100, and 300 mg kg^−1^, respectively, compared to acetic acid control.

### 4.6. Effect of Hydroethanolic Extract of *Cordia vignei* Leaves on Levels of TNF-*α* and IL-6 in the Serum of Acetic Acid-Induced Colitic Rats

#### 4.6.1. TNF-*α* Concentration

**Figure 6:**
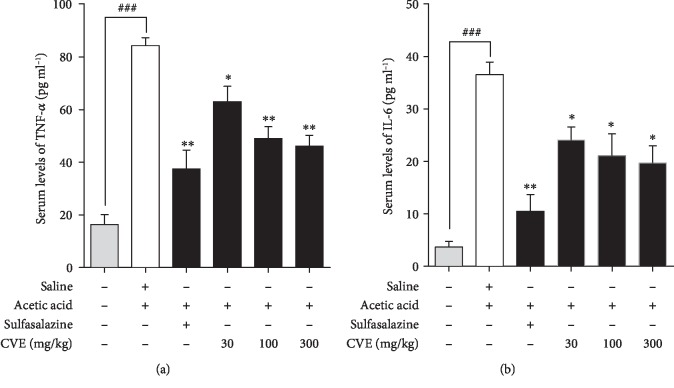
Effect of *Cordia vignei* leaf extract on serum levels of TNF-*α* and IL-6 in acetic acid-challenged rats. Rats (*n* = 5) were orally treated with normal saline (10 ml kg^−1^), sulfasalazine (500 mg kg^−1^), or CVE (30-300 mg kg^−1^) daily for 7 days. On day 4, rats were challenged with 500 *μ*l of acetic acid (4% *v*/*v*). Rats were sacrificed on day 8 and serum levels of TNF-*α* (a) and IL-6 (b) were assessed by ELISA. Results were presented as mean ± SEM. ^###^*P* < 0.001 (acetic acid control *vs.* naïve control); ^∗^*P* < 0.05, ^∗∗^*P* < 0.01 (acetic acid control *vs*. treatment group).

The TNF-*α* concentration in the serum of naive control rats was 16.34 ± 3.79 pg ml^−1^ (Figure 6(a)). Acetic acid significantly increased serum TNF-*α* concentration (pg ml^−1^) to 84.32 ± 3.06 (*P* = 0.0002). Sulfasalazine significantly reduced this TNF-*α* level (pg ml^−1^) to 37.40 ± 7.24 (*P* = 0.0040). Treatment of rats with CVE significantly reduced serum TNF-*α* concentration (pg ml^−1^) to 63.02 ± 5.77 (*P* = 0.0311), 48.94 ± 4.57 (*P* = 0.0030), and 46.03 ± 4.26 (*P* = 0.0019), respectively, at doses 30, 100, and 300 mg kg^−1^ (Figure 6(a)).

#### 4.6.2. IL-6 Concentration

The mean concentration of IL-6 in serum of the naïve control group was 3.68 ± 1.07 pg ml^−1^ and was significantly increased to 36.55 ± 2.40 (*P* = 0.0002) in the acetic acid control rats (Figure 6(b)). Sulfasalazine significantly reduced this concentration to 10.47 ± 3.23 (*P* = 0.0029) while CVE significantly reduced the serum concentrations of IL-6 to 24.12 ± 2.47 (*P* = 0.0225), 21.10 ± 4.15 (*P* = 0.032) and 19.72 ± 3.26 (*P* = 0.0142), respectively, at 30, 100, and 300 mg kg^−1^ (Figure 6(b)).

### 4.7. Effect of Hydroethanolic Extract of *Cordia vignei* Leaves on Haematological Parameters of Acetic Acid-Induced Colitic Rats

All the blood parameters of the naïve control rats were within the range of normal blood values of healthy rats (Table 1). Intrarectal administration of acetic acid did not cause any significant change in blood values compared to the naïve control. Sulfasalazine did not cause any significant change in all the blood parameters that were assessed. CVE caused a significant (*P* < 0.0077) reduction of lymphocyte level at 100 mg kg^−1^ albeit insignificant at 30 or 300 mg kg^−1^ compared to acetic acid control.

Also, CVE significantly (*P* < 0.05) reduced neutrophil levels at 100–300 mg kg^−1^, albeit, insignificant at 30 mg kg^−1^ compared to acetic acid control.

Male Sprague Dawley rats (*n* = 5) were orally treated with either normal saline (10 ml kg^−1^), sulfasalazine (500 mg kg^−1^), or CVE (30-300 mg kg^−1^) from day 0 to day 7. On day 4, colitis was induced as described in methods. Rats were euthanized on day 8 and blood samples were collected from the jugular vein for full blood count using automated cell analyzer. Mean blood values were statistically compared using *t* test. Results were presented as mean ± SEM. ^∗^*P* < 0.05 and ^∗∗^*P* < 0.01 (acetic acid control vs. treatment group).

## 5. Discussion

Ulcerative colitis experimentally induced by intrarectal administration of low concentration (usually 3-5%) of acetic acid is a well-recognized model for the study of inflammatory bowel disease [1]. Though acetic acid-induced ulcerative colitis and human inflammatory bowel disease may differ in aetiology, the two diseases share common pathophysiological features as well as sensitivity to drug treatment. For example, colonic changes such as mucosal ulceration, weight loss, hemorrhage, and inflammation which occur following intrarectal administration of acetic acid in rodents are also common in human IBD [18]. Also, influx of inflammatory cells such as neutrophils into the injured colon, rupture of colonic barrier, release of inflammatory mediators such as cytokines, arachidonic acid metabolites, and production of reactive oxygen species (ROS) which results in oxidative damage are common in both diseases [6].

In this study, intrarectal administration of acetic acid significantly reduced the body weight of rats. Loss of weight in colitis is due to deficiency of nutrients resulting from reduced appetite, food aversion or malabsorption, and rapid loss of body fluid through colorectal bleeding and diarrhoea. Also, TNF-*α* and IL-6 contribute immensely to loss of body weight in colitis by releasing neuropeptides that suppress appetite and precipitate cachexia [19]. Though serum levels of both TNF-*α* and IL-6 were significantly reduced by sulfasalazine and the extract, loss of body weight was not significantly affected by both agents in this study. Therefore, the weight loss could result from loss of body fluid through diarrhoea and bleeding.

Macroscopic examination of the colon revealed a significant increase in colon weight/length ratio of rats. This is due to severe tissue oedema, necrosis, goblet cell hyperplasia, and inflammatory cell infiltration [20, 21]. The macroscopic examination of intestinal content of CVE-treated rats showed well-formed fecal pellets without visible blood or mucus stains, and this could be due to uncompromised mucus layer and inhibition of excessive blood loss which are indicative of therapeutic success of potential antiulcerative agents [22–24]. The mucus layer is well known to enhance the repair of the chemically induced epithelial damage and also prevents diarrhoea and loss of blood through faeces [25]. It is therefore not surprising that preservation of mucus layer by CVE ameliorated colonic ulceration and reduced inflammatory score.

Histopathological assessment revealed that treatment of rats with *Cordia vignei* extract preserved the functional cytoarchitecture of the entire colonic mucosa and inhibited inflammatory cell infiltration, congestion, ulceration, erosions, necrosis, and hyperplasia caused by acetic acid. This is also indicative of the ability of the extract to protect the animals and reduce progression of the disease.

In healthy rats, superoxide dismutase (SOD) and catalase (CAT) play important roles as protective antioxidant enzymes. In ulcerative colitis, levels of these enzymes in colonic tissues become exhausted as a consequence of oxidative damage caused by free radicals [26]. SOD protects the cells against ulcerative damage by mediating dismutation of superoxide anion and preventing lipid peroxidation. SOD also prevents leukocyte rolling and adhesion in colonic tissues [27]. CAT, which is concentrated in subcellular organelles of peroxisomes, catalyzes the conversion of hydrogen peroxide, a cytotoxic compound to water and oxygen [27]. Malondialdehyde (MDA) is a byproduct of lipid peroxidation occurring in the tissue. In ulcerative colitis, levels of MDA in the plasma increases significantly and this is used as important diagnoses of patients with inflammatory bowel disease [6]. Since lipid peroxidation occurs during oxidative stress, natural products with antioxidant activity are beneficial [28]. Significant inhibition of MDA levels and increased SOD and CAT activities in the colons by CVE is indicative of its antioxidant potential which is important in its anti-inflammatory effect.

Tumor necrosis factor alpha (TNF-*α*) and interleukin 6 (IL-6) play essential roles in the pathophysiology of inflammatory bowel disease [29, 30]. They modulate mucosal immune system, alter epithelial integrity, and orchestrate neutrophil and macrophage infiltration and activation which result in colonic injury. TNF-*α* is involved in increased endothelia cell permeability, pyrexia, algesia, cachexia, and leukocyte production and activation to generate more prostaglandins (PGs). IL-6 stimulates production of acute-phase proteins and activation of the complement system thereby releasing C3 and C5. C5 and C3 are involved in neutrophil chemotaxis and activation of leukocytes to release more mediators and activation of mast cells to release histamine and heparin. Recently, TNF-*α*, IL-6, COX-2, and their upstream signal regulator, NF-*κ*B, have become new promising anti-inflammatory targets for the treatment of IBD [31]. In this study, elevation of TNF-*α* and IL-6 levels in the control rats as opposed to the reduced levels in the CVE-treated rats is suggestive of antiulcerative potential of the extract. Also, treatment of rats with CVE which significantly inhibited TNF-*α* and IL-6 elevation and prevented depletion of SOD and CAT levels resulted in gross protection of colons.

These findings are similar to those reported by Aleisa et al. [1], Gautam et al. (2012) [2], Lopes et al. (2014) [32], and Nartey et al. [33] in which *Gymnema sylvestre*, *Antrocaryon micraster*, *Terminalia chebula*, *Solanum cernuum*, and *Cassia sieberiana* extracts attenuated acetic acid-induced ulcerative colitis in rats by inhibiting various physical, haematological, and biochemical markers such as body weight loss, colon density, TNF-*α* and IL-6 expressions, neutrophil infiltration, and oxidative stress although in this research, loss of body weight was not significantly affected by CVE.

## 6. Conclusion

The findings of the present study are indicative of the preventive ability of *Cordia vignei* leaf extract against the damaging effect of acetic acid-induced colitis in Sprague Dawley rats through inhibition of TNF-*α* and IL-6 activities. Also, SOD and CAT activities in the colons were increased. The results may be useful in future clinical trials of the *Cordia vignei* leaves or its bioactive constituents as natural, safe and effective treatment of patients with inflammatory bowel disease.

## Figures and Tables

**Table 1:** Effect of hydroethanolic leaf extract of *Cordia vignei* on haematological parameters of acetic acid-induced colitic rats.

Treatment (group)	WBC (×10^3^/*μ*l)	RBC (×106/*μ*l)	HGB (g/dl)	LYMP (%)	NEUT (%)	HCT (%)	PLT (×103/*μ*l)
Nonacetic	11.32 ± 1.03	10.56 ± 095	14.51 ± 0.77	69.5 ± 3.33	7.5 ± 0.57	45.72 ± 4.32	593 ± 14.38
Acetic acid	13.47 ± 0.97	8.87 ± 1.18	13.61 ± 0.89	57.8 ± 3.96	8.9 ± 1.12	42.4 ± 1.80	631 ± 20.01
Sulfasalazine	12.71 ± 1.02	6.95 ± 0.88	12.92 ± 1.25	51.9 ± 2.96	6.7 ± 0.69	49.31 ± 2.89	583 ± 36.88
30 mg/kg CVE	10.32 ± 1.07	7.46 ± 0.64	11.95 ± 0.86	61.7 ± 6.26	7.3 ± 0.45	48.66 ± 4.58	667 ± 20.77
100 mg/kg CVE	11.61 ± 1.52	6.71 ± 0.67	12.94 ± 1.00	42.2±1.94^∗∗^	5.7 ± 0.46^∗^	43.12 ± 2.50	580 ± 17.01
300 mg/kg CVE	12.14 ± 0.99	7.18 ± 0.68	13.67 ± 0.90	47.3 ± 2.34	6.2 ± 0.16^∗^	44.90 ± 1.79	624 ± 16.40

## Data Availability

This study forms part of the extensive research on health benefits of hydroethanolic extract of Cordia vignei leaf for the award of Doctor of Philosophy at Kwame Nkrumah University of Science and Technology, Kumasi, Ghana. The data shall be deposited at the Research Repository at the University's Library (http://www.knustspace.com/) and could be accessed through the corresponding author upon request.

## Supplementary Materials

Supplementary Materials Phytochemical screening and Fourier-transform infrared (FTIR) spectroscopic analysis of hydroethanolic extract of Cordia vignei leaves [34–36].
