# Supplementary material for: Acetic Acid-Induced Ulcerative Colitis in Sprague Dawley Rats Is Suppressed by Hydroethanolic Extract of Cordia vignei Leaves through Reduced Serum Levels of TNF-α and IL-6
Source: Int J Chronic Dis. 2020 Feb 6;2020:8785497. doi: 10.1155/2020/8785497 (PMC7026722; doi:10.1155/2020/8785497)
Supplement: Supplementary Materials — Phytochemical screening and Fourier-transform infrared (FTIR) spectroscopic analysis of hydroethanolic extract of Cordia vignei leaves [34–36]. [file 8785497.f1.pdf]

Supplementary Data

Materials and Methods

Materials

Ferric chloride, conc H_2SO_4, Potassium bismuth iodide solution, chloroform (all chemicals were supplied by Department of Pharmacognosy, KNUST, Kumasi Ghana).

Method

Fresh leaves of *Cordia vignei* were collected and processed to obtain the extract as described in section 2.1.

1. Phytochemical screening of hydroethanolic extract of *Cordia vignei* leaves.

The extract was screened qualitatively for the presence of phytochemicals such as tannins, saponins, glycosides, steroids, alkaloids, coumarins, flavonoids and terpenoids by using simple protocols as described by Sofowora, (1993) [34].

2. Fourier transform infrared (FTIR)-spectroscopic analysis of the extract

The Fourier transform infrared spectroscopy was conducted to detect the characteristic functional groups of the hydroethanolic extract of *Cordia vignei* leaves using the procedure previously described by Pavia *et al.,* (2001) [35] and Lakshmia *et al.,* (2015) [36]. Briefly, 5 mg of the extract was pressed with potassium bromide (KBr) and the pellet was analyzed by using Spectrum Two FT-IR Spectrometer (PERKIN-ELMER, UATR2, 94133, USA).

Results

1. Phytochemical screening of the extract

The qualitative phytochemical test showed that saponins, tannins, alkaloids, flavonoids and terpenoids were present in the extract.

2. Fourier transform infrared (FTIR) spectroscopy

The Fourier transform infrared (FTIR) spectra are shown in the figure S1 below. The characteristic functional groups representing these peaks (cm^{-1}) are presented in table S1. Wavenumbers in the fingerprint region (1500-400 cm^{-1}) and their functional groups were not shown on the table.

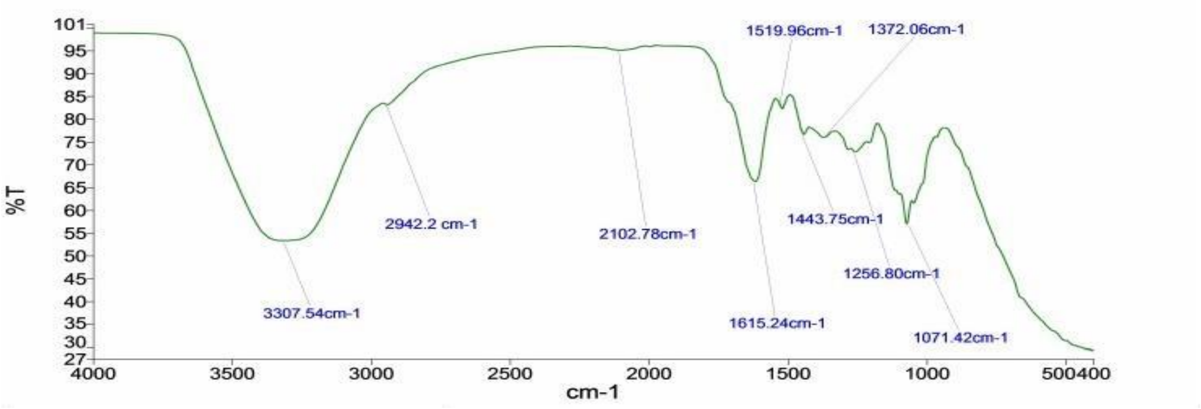

Figure S1. Fourier transform infrared spectra of hydroethanolic extract of *Cordia vignei* leaves. Five milligram (5 mg) of the extract was pressed with KBr to form a disc and this was analyzed by using Spectrum Two FT-IR Spectrometer. The results were presented as wavenumber (cm^{-1}) (horizontal) against percentage transmittance (% T) (vertical). The wavenumbers and their corresponding functional groups are presented in the table S1 below.

Table S1. Characteristic functional groups identified in FTIR spectroscopy

number	Wavenumber (cm^{-1})	Functional groups
1	3307.20, 1256.80	Phenol
2	3307.20, 1071.42	Alcohol
3	2933.69	Alkane
4	1615.24	Alkene
5	1517.93	Aromatic compound

Five milligram (5 mg) of the extract was pressed with potassium bromide (KBr) to form a disc and this was analyzed by using Spectrum Two FT-IR Spectrometer. The results were presented as wavenumber (cm^{-1}) (horizontal) against percentage transmittance (% T) (vertical) as shown on figure S1 above.
